# Medical and Philosophical Intelligence

**Published:** 1816-01

**Authors:** 


### MEDICAL AND PHILOSOPHICAL INTELLIGENCE.

### KING *v.* TAUNTON.

AS the event to which the following prosecution refers will probably never occur again, we have thought it desirable to preserve a record of the form of such an indictment, which, we hope, may at least entertain our readers. It may also instruct them in the construction of a law hitherto imperfectly understood. We shall only make one remark on legal tautology, viz. that, instead of rendering a subject more precise and distinct, it seems to us, who are accustomed to medical accuracy, to confound all distinction: words, which by degrees have found their appropriate meaning, being used as synonimous.

"*Of Trinity Term, in the Fifty-fifth of*
Middlesex.] *King George the Third.*

Be it remembered, that on Tuesday next after three weeks from the day of the Holy Trinity, in the fifty-fifth year of the reign of our Sovereign Lord George the Third, by the Grace of God, of the United Kingdom of Great Britain and Ireland, King, Defender of the Faith, in the county of our said Lord the King, before the King himself, at Westminster, in the county of Middlesex, By the oath of twelve jurors, good and lawful men of the said county of Middlesex, now here sworn and charged to enquire for our said Lord the King, for the body of the said county, It was presented as followeth (that is to say):

Middlesex—The jurors for our Lord the King, upon their oath, present that *John Taunton*, late of the parish of St. Andrew, Holborn, in the county of Middlesex, surgeon, on the twenty-third day of March, in the fifty-fifth year of the reign of our Sovereign Lord George the Third, by the grace of God of the United Kingdom of Great Britain and Ireland, King, Defender of the Faith, and on divers other days and times between that and the tenth day of June, in the same year, with force and arms, at the parish aforesaid, in the county aforesaid, unlawfully and injuriously did inoculate and infect Edgar Cooke, an infant of tender age, to wit, about the age of one year; George Benman, an infant of tender years, to wit, about the age of three years; Eliza Briggs, an infant of tender age, to wit, of the age of fifteen months; and divers other infants of tender years, whose names to the jurors aforesaid are unknown, with a certain contagious, infectious, and dangerous sickness and disease, called the small-pox, by means whereof the said Edgar Cooke, George Benman, William Luckett, Eliza Briggs, and the said divers other infants, on the said twenty-third day of March, in the year aforesaid, and on the said other days and times at the parish aforesaid, in the county aforesaid, became and were ill and sick of and with the said contagious, infectious, and dangerous sickness and disease: And that the said John Taunton, well knowing the premises aforesaid, after he had so inoculated and infected the said Edgar Cooke, George Benman, William Luckett, Eliza Briggs, and the said divers other infants, and whilst the said Edgar Cooke, George Benman, William Luckett, Eliza Briggs, and the said divers other infants, were ill and sick of and with the aforesaid contagious, infectious, and dangerous sickness and disease as aforesaid, to wit, on the said twenty-third day of March, in the year aforesaid, and on the said other days and times, with force and arms, at the parish aforesaid, in the county aforesaid, unlawfully and injuriously did cause and procure the said Edgar Cooke, George Benman, William Luckett, Eliza Briggs, and the said divers other infants, to be taken, carried, and conveyed into and along a certain public street and common highway called Hatton Garden, situate and being in the parish aforesaid, in the county aforesaid, and into and along divers other public streets and common highways there also situate, and being used for all the liege subjects of our said Lord the King, on foot, and with horses, coaches, carts, and carriages, to go, return, pass, ride, and labour, in, along, and through; in and along which said several streets and common highways these divers liege subjects of our said Lord the King were then, to wit, on the said twenty-third day of March, in the year aforesaid, and on the said other days and times, going and returning, passing, riding, and labouring; and near unto and by divers dwelling-houses, habitations, and residences of divers liege subjects of our said Lord the King, then and there dwelling, inhabiting, and residing, to wit, on the said twenty third day of March, in the year aforesaid, and on the said other days and times, at the parish aforesaid, in the county aforesaid, to the great danger of infecting with the said contagious, infectious, and dangerous sickness and disease called the small-pox, all the liege subjects of our said Lord the King, who, on the several days and times aforesaid, were in and near the aforesaid public streets and common highways, dwelling-houses, habitations, and residences, and who had not had the said disease and sickness: to the great damage and common nuisance of all the last-mentioned liege subjects of our said Lord the King, to the evil example of all other persons in like cases offending, and against the peace of our said Lord the King, his crown, and dignity.

Second count.—And the jurors aforesaid, upon their oath aforesaid, do further present that the said John Taunton, on the said twenty-third day of March, in the fifty-fifth year aforesaid, with force and arms, at the parish aforesaid, in the county aforesaid, unlawfully and injuriously did inoculate and infect the said Edgar Cooke with the aforesaid contagious, infectious, and dangerous sickness and disease called the small-pox, by means whereof the said Edgar Cooke, on the said twenty-third day of March, in the year aforesaid, and continually afterwards from thence until the third day of May, in the same year, at the parish aforesaid, became and was ill and sick of and with the said contagious, infectious, and dangerous sickness and disease; and that the said John Taunton, well knowing the premises last aforesaid, after he had so inoculated and infected the said Edgar Cooke, as last aforesaid, and whilst the said Edgar was ill and sick of and with the aforesaid contagious, infectious, and dangerous"—repeating the same words as in the former count, and concluding with "the evil example of all other persons in like cases offending, and against the peace of our said Lord the King, his crown, and dignity."

The third count repeats the same relative to George Burman.

Fourth count recounts the same of William Luckett, in the same words.

Fifth count recounts the same of Eliza Briggs—the whole of the indictment occupying fifteen folio pages of lawyer's sheets.

Mr. Taunton vaccinated his own children, was one of the first subscribers to the London Vaccine Institution, and has been constantly on the board of managers of that charity: at the same time he felt it his duty to inoculate such for the small-pox who, through prejudice or otherwise, refused vaccination. Many of the poor who applied for gratuitous advice, applied also for inoculation for the cow-pock, and some for the small-pox. No preference or recommendation was given to either—for vaccination the matter is always obtained from Dr. John Walker. Such was Mr. T.'s practice till the 11th of June, when the report of Burnett's sentence was published, *from which day* no person applied for inoculation who did not receive a copy of the following notice:—"You are desired to return in seven or eight days, to say how the child is, but on no account to expose it to other children: if required, it will be visited." On the 19th June, Mr. Taunton was arrested on the Lord Chief Justice's warrant: he gave bail, and directed his attornies to defend the cause, which was to have been tried on Friday, Dec. 8th, in the Court of King's Bench, where Mr. Taunton attended with his witnesses. Sir William Garrow, the attorney-general, and counsel for the plaintiff, stated to the court that he should not proceed in the present case, as he learnt that the defendant had given notice, with every inoculation, not to expose their children while the disease was out, and that *this had been done from the time that the sentence of that court in the case of Burnett had been known.*

The opinion given by the court was, that all persons were at liberty to inoculate, or to have their children inoculated, for the small-pox; but to expose publicly children or others while the disease was out full upon them, was an indictable offence.

Mr. Taunton always declared his determination of trying the question, in defence of his practice; but on the day of trial his solicitor expressed himself thus: "The Attorney-general seems to have been aware of the *real objects* of the prosecution, and on the behalf of his client to have made the most polite retreat in his power, but which we could not possibly *prevent.*"

From the reports in the various newspapers, it would seem that inoculation for the small-pox had been discontinued from the time of the present prosecution:—that is not true; patients are inoculated lated now, and have been as at all former periods, but none are allowed to go abroad with the disease out upon them.

The persons most conspicuous in the information and prosecution were Mr. Barnett and Mr. Charles Murray: the former, surgeon in Smithfield Bars, who must have been under some erroneous impressions, as he has often obtained small-pox matter from Mr. Taunton's patients; the latter is a *lawyer* in Sun-court, Cornhill, and holds an office of some kind in the National Vaccine Institution.

---

*Royal Society.*—On St. Andrew's Day, this Society held their annual meeting, at their apartments in Somerset-place, when the President, the Right Hon. Sir Joseph Banks, in the name of the Society, presented the gold medal (called Sir Godfrey Copley's) to David Brewster, LL.D. for a paper "on the Polarisation of Light by Reflection from Transparent Bodies," printed in the last volume of the Philosophical Transactions. Afterwards the Society proceeded to the choice of a Council and Officers for the ensuing year, when, on examining the lists, it appeared that the following gentlemen were elected:—

*Of the Old Council.*—The Right Hon. Sir Joseph Banks, Bart. K.G.C.B; Sir Charles Blagden, Knt.; Samuel Goodenough, Lord Bishop of Carlisle; Taylor Combe, Esq.; Davies Giddy, Esq. M.P.; Sir Everard Home, Bart.; Samuel Lysons, Esq.; George, Earl of Morton, K.T.; John Pond, Esq.; William Hyde Wollaston, M.D.; Thomas Young, M.D.

*Of the New Council.*—John Barrow, Esq.; Mark Beaufoy, Esq. Henry Browne, Esq.; Sir Humphry Davy, Knt.; Philip, Earl of Hardwicke, K.G.; Edward Howard, Esq.; John Latham, M.D. Pres. Col. Phys.; Thomas James Mathias, Esq.; Sir John Nichol, Knt. M.P.; George, Earl of Winchelsea, K.G.

*Officers.*—President, the Right Hon. Sir Joseph Banks, Bart. K.G.C.B. Treasurer, Samuel Lysons, Esq. Secretaries, William Hyde Wollaston, M.D. and Taylor Combe, Esq.

After the election, the members of the Society dined together, as usual, at the Crown and Anchor Tavern in the Strand.

---

*Royal Society of Sciences of Copenhagen.*—Mr. Wad, Chancellor of State, exhibited an elegant and faithful model in wax, of a very rare *amphibium*, called Proteus *anguinus*; a piece of *aerolite*, containing *iron* and *nicolum*, which had been long preserved in the town-house of Elbogen, in Bohemia; and a new fossil *(gurtrofian)*, consisting of calx carbonica and talcum carbonicum.

---

*Royal Medical Society of Copenhagen.*—Mr. Wendt has presented two memoirs; one upon *antimonium tartarizatum*, and the different ways of preparing it; the other, on the exhibition of the root of the *cucubalus viscosus* as a medicine, and its operation upon the human system.—Mr. Jacobson also read a paper upon the noxious effects of bitter almonds.

One

One of the German journals, some time ago, announced the death of the celebrated SCARENGER, by the application of prussic acid to his naked arm. Before this, the Batavian Society, after a repetition of several of Mr. Brodie's experiments, had concluded with very different results. We were unwilling, therefore, to insert either, till we could gain better information. It now appears that valuable character died apoplectic.

---

PRIZE QUESTIONS.—The *Cercle Médical de Paris* had offered the following queries as the subjects of a prize dissertation for 1814; but having received no essay that was satisfactory, the period was extended to 1815: and now again, for a similar reason, it is protracted to 1817.

1. What is the nature of the disease known under the name of Rabies *(de rage)?* 2. What are the symptoms which characterize it in man and in the lower animals? 3. Is it ever spontaneously developed in man? 4. Are there many species of it, and what are these? 5. Are they all contagious in man, and how are they communicated? 6. Whether should the morbid effects which follow the bite of a rabid animal be attributed to a particular virus, or to the nature of the bite, or to the physical lesion of the wounded parts, or to terror? 7. What is the best mode of treatment, both prophylactic and curative.

The prize, which is a gold medal worth 300 francs, shall be adjudged at an extraordinary public sitting of the society in March 1817. The dissertations to be written in French or Latin, with a motto; and to be transmitted, free of postage, with a sealed cover containing the name of the author, to M. le Docteur Chardel, Secretaire Genéral du Cercle Médical, rue Cassette, No. 23, à Paris, before the end of 1816.

*The Royal Academy of Sciences, Belles-Lettres, and Arts, of Rouen*, having received no essay on the question which it proposed for 1814, and continued for 1815, have withdrawn it; and now propose the following as the subject of a prize dissertation for 1816:

"To show, abstracted of every hypothesis, the consequences which naturally result from the observations and experiments hitherto made relative to the motion of the sap in plants; to confirm these results by new observations and experiments; and to point out how the facts already known regarding the movements of vegetable fluids may be rendered useful to the art of cultivation?"

The prize, a gold medal worth 300 francs, will be adjudged in the public sitting of 1816. The essays, written in French or Latin, must be sent, free of postage, to M. Vitalis, Perpetual Secretary of the Academy, before the 1st of June, 1816.

*The Society of Sciences at Haerlem* has also proposed the following as subjects of prize dissertations:

I. How far has chemistry developed the ultimate and proximate constituent principles or parts of plants, particularly of alimentary plants; and how far can we ascertain, from the knowledge we possess on this subject, which plants are the most salutary to the human body in the state of health, and in some diseases?—II. As the antiseptic property of sea-salt does not appear to depend solely on the muriate of soda, but also on the muriate of magnesia which is mixed with common salt; it is required to determine by experiment—1. Which of these salts is the most powerful antiseptic? 2. What is the proportion in which the two salts ought to be combined to resist, for the longest time, the putrefactive process, without communicating a disagreeable taste to the substances they are intended to preserve? 3. Can muriate of magnesia alone be used in any case, particularly in voyages to warm climates? 4. Can salt-petre works be established with advantage in places where the water is impregnated with the products of putrefaction? 5. What are the causes of the contagious diseases which prevail in besieged places? and what are the best means which our physical and chemical knowledge indicate for their prevention or destruction? A history of these diseases is not required to be given, neither is it necessary to state the modes of treating them, but an exposition, founded on experiments, of their causes; and particularly the physical and chemical means requisite for overcoming them. 6. Are acid fuminations, such as those of muriatic acid, and particularly of the oxymuriatic acid, the great efficacy of which is well known, always sufficient, in every case, for destroying the miasma, or morbific matter spread in the atmosphere: or is the opinion of some physicians more correct, that under certain circumstances, instead of the acids or oxydizing matters, alkaline or deoxydizing substances, such as ammonia and sulphurous acid, ought to be employed? 6. The practice of agriculture having proved that, in the first stage of the vegetation of corn, and other plants cultivated until they flower, the fertility of the ground is scarcely diminished, although, after the fructification and maturity of the grains, the same soil is considerably impoverished and robbed of its fecundity: what is the cause of this phenomenon, and how far would the solution of it furnish rules, the practice of which would perfect the culture of the soil? 7. As atmospheric air is sooner rendered unrespirable by smoking coals than by ardent coals, although the latter produce a larger proportion of carbonic acid gas; the society is desirous of having the change which atmospheric air sustains from smoking coals examined, and compared with that which is produced by ardent coals, so as to ascertain the cause of the sudden asphyxias occasioned by it. 9. As the chemical analysis of vegetables is not yet arrived at the degree of perfection desirable, the society offers the double gold medal, worth 300 Dutch florins, to any one who, by new experiments, shall carry the analysis of plants to the highest degree of perfection.

The prize offered for each of these questions is a gold medal, or 150 Dutch florins, whichever the author may prefer: and the society ciety will be better pleased if the authors who may be candidates forbear from loading their essays with extraneous matter, and adhere strictly to the question.

The dissertations may be written in Dutch, French, Latin, or German, and should be transmitted, accompanied with a sealed cover inclosing the name of the author, before the 1st of January 1816, to M. Van Marum, Perpetual Secretary of the Society of Haerlem.

*The University of Copenhagen* proposed the following prize question for the present year:

Quaenam est aquæ puræ frigidæ vis in sanitatem tuendam et morbos profligendos?

---

A French journal speaks of milk as a remedy for the poison of certain mushrooms. Before we trust to such a remedy, several experiments must be made on other animals, with such mushrooms, exhibited with and without milk.

---

*Craniology.*—Mr. Forster has of late been making some observations on the heads of insane persons in some public madhouses, with a view to determine how far the particular organization of the head appears to modify the character of the mania. He has found in the majority of cases a very striking connexion, particularly with regard to the melancholic insane, who have been found to have the parts of the brain which, according to the new system, produce the sentiment of fear, much developed. It is to be hoped that repeated observations of this kind may lead to important discoveries in the history of this interesting, but at present obscure, class of disorders.

Dr. Spurzheim has given an introductory lecture on the new anatomy to a scientific class of above 300 persons at Dublin.

---

A physician at Carlsruc has been repeating the exploded acids in syphilis, and, of course, with success. Can we blame our neighbours, when we reflect on our own past folly, who had the advantage of Mr. Hunter's accuracy to direct us better.

---

The following case can only be accounted for on the explanation of Gall and Spurzheim, as adopted by Sir Everard Home.

A case of chronic hydrocephalus occurred in the practice of Dr. Read of Kilmarnock, which shows the great changes that may take place in the brain, without materially impairing the functions of that organ. The subject of the disease was a boy five years of age, in whom an enlargement of the head was first perceived when he was three months old, which increased until his death (produced by an epidemical disorder), at which period the dimensions of the head were as follows:—Round the head, by the brow and occiput, 29½ inches. From ear to ear, over the forehead, 19 inches. Round the head, by the chin and vertex, 31 inches. Thirty-four gills (8½ pints) of water were contained within the head. When the the water had all flowed out, the cranium resembled a large case thick lined; the cortical and medullary portions of the brain were squeezed together, and formed what seemed the lining of the cavity, and was about one-fourth of an inch in thickness all round. A few shreds appeared extending through it, in the direction of front and occiput, being apparently the remains of the partition between the ventricles. The rest of the cerebrum was absorbed: the cerebellum was seen lying at the bottom, healthy and entire. Notwithstanding the early existence of this disease, and its rapid progress, it appears that this child's faculties expanded faster than is usual in children of his age; his vision was perfect; his hearing acute; his memory quick and tenacious; and his correctness in his childish affairs very remarkable. His senses were perfect till within an hour of his death.—*Edin. Journ.*

---

Our continental correspondents have sent us a number of articles, from which we have selected the most interesting. The long paper on Cartilaginous and Osseous Bodies found in the Joints is only a very imperfect transcript of Mr. Hunter's paper in the Transactions of a Society for the Improvement of Medical and Chirurgical Knowledge, and also of Mr. Brodie's paper on the same subject in the Medico-Chirurgical Transactions. On the subject of curing syphilis without mercury, and also without ascertaining the disease, our communications are innumerable. We have selected a few as specimens of the rest.

Dr. Karpe, at Vienna, has, on numerous occasions, experienced the efficacy of the diluted Acidum Sulphuricum, in the proportion of six drachms to two and a half of water, applied as a lotion in cases of typhus. He has observed, that, by this lotion, the stadium irritationis was not unfrequently quite suppressed; but that even applied at a later period it not only greatly alleviated the accidents of the typhus, but also accelerated the re-convalescence: many German physicians have confirmed this observation.

Prof. Meyer and Reich, at Berlin, now apply, as a principal remedy in cases of diarrhœa, so frequent in children, ℈j. acid. mur. dilut. in ℥iij. of any kind of syrup, to be taken a tea-spoonful every one and half or two hours.

Prof. Meyer affirms his having found great benefit in the fatal tympanites, particularly in nervous complaints, from a composition of alum, 8 or 10 grains, and 8 grains of the pulvis aromaticus, to be taken every two hours, combined with glysters of Acidum salis.

Dr. Frank has revived the practice of Urtication in those cases in which the power of a limb has ceased, from long disuse. He mentions the case of a soldier whose lower extremities were struck with the palsy, in all probability contracted by sleeping on the wet ground. I chose (said he) particularly the large stinging nettle for that purpose, of which I caused a considerable bundle to be tied together, and to be applied on the lower parts, upwards and downwards, as long as the patient could bear it, to which the use of warm baths is to be added. But in cases of palsy, the consequence of an apoplexy or hemiplegy, I could never procure any effect by urtication.

We print the two following articles to show how slow the progress of every art remains, till some luminary appears, to direct us to the proper road. How much soever we may smile at our neighbours, we ought to recollect that before Mr. Hunter's time we reasoned like them, and are only now beginning to reform:—

For some time past the Veterinary Institute at Vienna has made use of mercurial ointments in such diseases of animals as originate from the corruption of the lymphatic system. It has been discovered, by a lucky chance, that the use of the milk of such cows as had received the strongest unction, is an excellent remedy in venereal complaints. Repeated trials have confirmed the efficacy of this remedy in the most inveterate syphilitic complaints, although, after various decompositions, even of greater quantities of the milk, not the smallest visible particle of mercury could be discovered, notwithstanding large balls were found in the vessels of the animals to whom it had been given.

Dr. Rosenbaum found the gargling with the fresh juice of the herb chelidonium very beneficial in venereal ulcers of the throat.

---

*A Case of Demonomania, detailed by M. Bertholet, Médecin des Armees.*—The subject was a female, and the occasion of the disease was a sermon delivered by a most violent fanatic. Bertholet saw this woman on the fourth day of the disease. He found her in the highest state of most deplorable mental excitement, nor was it till he assured her that he was thoroughly initiated in the secrets of hell, that she would pay him any attention; but, upon hearing this, she said that violent pain existed in her head. A bath was proposed: it was objected to, because it would increase the flame consuming her; it was, however, permitted afterwards, as it would satisfy him of her real condition, by rendering the fire in her apparent. Extreme cold was applied to the head, by means of snow in a bladder. This was immediately followed by alleviation of symptoms, and its re-application was always attended with temporary good effect. On the fifth day she was exorcised, after the ceremonials of the Catholic church. Snow continued—its use remitted, violent delirium supervened—it was re-applied with benefit; purges were given, and opiates. A violent thunder clap at once operated a cure. The patient became calm, was wretched that she had behaved so ill, and gradually was restored to her usual health.—*Jour. de Phys. Feb.* 1815.

---

The following Memoir on partial Amputation of the Foot, by L. R. Villermé, D.M.P. contains an account of Chopart's operation, first published in 1792, which has for its object preserving the the posterior part of the foot, by amputating at such articulations of the tarsus as circumstances admit of. The operation was followed by a partial re-union, from the first intention, of the two flaps which were formed, to the articulating surfaces of the bone, and complete cicatrization occurred at the end of a month by suppuration of the rest of the wound. Chopart deserves much credit for this operation. A most important part is preserved, by which, both standing and walking, are secured to the subject of incurable ulceration of the other part of the feet, and which, before this improvement, had always been most strikingly impeded by the usual operation, viz. amputation of the lower leg.

Chopart tells us so successful is this treatment, that the patient stands in no need of mechanical assistance afterwards, but can walk perfectly well without it. This, however, was not supported by the experience of Villermé: he says he was led to believe, at the first examination, if we could add to the remaining extremity of the foot another mechanical extremity, thus augmenting the base of support which has been diminished by the operation, we shall effect the cure of the disease, and supply the loss from amputation; for we thus replace in some measure, or form anew, the portion of the dismembered extremity. This artificial extremity will have all the perfection of which it is susceptible, if maintained in the direction of the foot by a strong interior spring.

It will be impossible in this article more than to mention the advantages Mr. Villermé urges in favour of the operation he recommends, or to point out in what it differs from that of Chopart. The author is astonished that the success of Chopart should not have led to new trials. It was not till 1812, that notice was taken of it by M. Richerand, in the *Nosographie Chirurgicale*, and in the Memoirs of Military Surgery by Mr. Larrey. The advantages proposed by Villermé appear to depend more particularly, 1st, on the remaining length of that portion of the foot which is anterior to its articulation with the leg; and on its size, which both better insure walking, and prevent falling forward. 2d, On the facility we shall have in supplying an artificial extremity to the foot, equal to the whole extent of the base of support, and the whole length of the column which forms the inferior limb.—*Ibid.*

---

Dr. Uwins is appointed Physician to the City Dispensary, vacated by Dr. Walker.

---

Dr. Henning, of the Hot-Wells, Bristol, author of an Inquiry into the Pathology of Scrofula, is preparing for the press a work on Pulmonary Consumption, which will be ready for publication early in the spring.

 Dr.

Dr. Adams will commence his Course of Lectures on the Institutes and Practice of Medicine, on Tuesday, January 13th, at ten o'clock precisely, at his house in Hatton Garden.—For further particulars see the cover.

---

*Theatre of Anatomy.*—The Winter Course of Lectures on Anatomy, Physiology, Pathology, and Surgery, by Mr. John Taunton, F.A.S. Member of the Royal College of Surgeons of London, Surgeon to the City and Finsbury Dispensaries, City of London Truss Society, &c. will commence on Saturday, January 27th, 1816, at eight o'clock in the evening precisely, and be continued every Tuesday, Thursday, and Saturday, at the same hour.

---

The Spring Courses of Lectures will commence at St. Bartholomew's Hospital on Saturday, January 20th.—On the Theory and Practice of Medicine, by Dr. Hue. On Anatomy and Physiology, by Mr. Abernethy. On the Theory and Practice of Surgery, by Mr. Abernethy. On Chemistry and Materia Medica, by Dr. Hue. On Midwifery, by Dr. Gooch. Anatomical Demonstrations, by Mr. Stanley.

---

*Russell Institution.*—Mr. Singer will commence his Lectures on Electricity, Galvanism, and Electro-Chemistry, about the end of January.

---

Dr. Clutterbuck will begin his Spring Course of Lectures on the Theory and Practice of Physic, Materia Medica, and Chemistry, about the middle of January, at ten o'clock in the morning, at his house, No. 1, in the Crescent, New Bridge-street.

---

Mr. Clarke will commence his next Course of Lectures on Midwifery, and the Diseases of Women and Children, on Wednesday, January 24th. The Lectures are read every morning, from a quarter past ten to a quarter past eleven, for the convenience of students attending the hospitals.

---

Dr. Squire will, on Tuesday, January 16th, begin a Course of Lectures on the Principles and Practice of Midwifery, including the Diseases of Women and Children.

---

Mr. Charles Bell will re-commence his Surgical Lectures on Tuesday evening, the 23d instant, at seven o'clock. He will give a Clinical Lecture on the cases in the Hospital every week during the course.

LONDON

### LONDON PRICES OF DRUGS.—December 22, 1815.

| | £. | s. | d. | £. | s. | d. | per |
|---|---|---|---|---|---|---|---|
| Aloes Barbadoes, from | 17 | 0 | 0 to | 18 | 0 | 0 | C. |
| —— Cape | 4 | 0 | 0.. | 4 | 5 | 0 | — |
| —— Succotrina | 23 | 0 | 0.. | 0 | 0 | 0 | — |
| —— Epatica or E.I. | 9 | 10 | 0.. | 0 | 0 | 0 | — |
| Angelica Root | none. | | | | | | |
| Alkanet Root | 6 | 10 | 0.. | 7 | 0 | 0 | — |
| Antimony Crude | 2 | 10 | 0.. | 2 | 14 | 0 | — |
| Arrow Root, fine | 0 | 2 | 0.. | 0 | 2 | 3 | lb. |
| —— ordinary | 0 | 0 | 9.. | 0 | 1 | 0 | — |
| Arsenic, Red | 4 | 4 | 0.. | 4 | 10 | 0 | C. |
| —— White | 2 | 16 | 0.. | 0 | 0 | 0 | — |
| Balsam Capivi | 0 | 4 | 6.. | 0 | 4 | 9 | lb. |
| —— Peru | 0 | 18 | 0.. | 1 | 1 | 0 | — |
| —— Tolu | 0 | 12 | 6.. | 0 | 13 | 0 | — |
| Bark Jesuits', pale ord. | 0 | 1 | 3.. | 0 | 0 | 0 | — |
| —— middling | 0 | 2 | 1.. | 0 | 0 | 0 | — |
| —— fine | 3 | 0 | 0.. | 0 | 0 | 0 | — |
| —— Crown | 0 | 3 | 8.. | 0 | 4 | 6 | — |
| —— Yellow flats | 0 | 1 | 2.. | 0 | 1 | 4 | — |
| —— Quills | 0 | 2 | 0.. | 0 | 2 | 3 | — |
| —— Red Quills | 0 | 6 | 6.. | 0 | 8 | 9 | — |
| Borax, refined E.I. | 6 | 0 | 0.. | 6 | 10 | 0 | — |
| —— English | 0 | 2 | 0.. | 0 | 2 | 3 | lb. |
| —— unrefined or Tinc. | 5 | 5 | 0.. | 0 | 0 | 0 | C. |
| Camphire, refined | 0 | 5 | 3.. | 0 | 0 | 0 | lb. |
| —— unrefined | 14 | 10 | 0.. | 15 | 0 | 0 | C. |
| Cantharides | 0 | 8 | 6.. | 9 | 0 | 0 | lb. |
| Cardemoms (best) | 0 | 5 | 3.. | 0 | 5 | 6 | — |
| Cassia Buds | 20 | 10 | 0.. | 23 | 0 | 0 | C. |
| —— Fistula, W.I. | 5 | 0 | 0.. | 0 | 0 | 0 | — |
| —— Lignea | 25 | 0 | 0.. | 26 | 0 | 0 | — |
| Castorum American | 1 | 8 | 0.. | 2 | 2 | 0 | lb. |
| —— Russia | 11 | 11 | 0.. | 0 | 0 | 0 | — |
| Castor Oil, per bottle 1½ lb. | 0 | 3 | 4.. | 0 | 3 | 9 | bo |
| Coculus Indicus | 3 | 3 | 0.. | 3 | 10 | 0 | C. |
| Colocynth Turkey | none. | | | | | | |
| Columbo Root | 3 | 0 | 0.. | 3 | 5 | 0 | C. |
| Cream of Tartar | 4 | 15 | 0.. | 5 | 0 | 0 | — |
| Gallangal East-India | 4 | 10 | 0.. | 4 | 15 | 0 | C. |
| Gentian Root | 4 | 15 | 0.. | 4 | 17 | 6 | — |
| Ginfang | 4 | 0 | 0.. | 4 | 3 | 0 | lb. |
| Grains of Guinea | 5 | 0 | 0.. | 5 | 10 | 0 | C. |
| Gum Ammo. Drop | 0 | 0 | 0.. | 0 | 0 | 0 | — |
| —— Lump | 10 | 0 | 0.. | 12 | 12 | 0 | — |
| —— Animi | 10 | 10 | 0.. | 0 | 0 | 0 | — |
| —— Arabic, E.I. | 2 | 0 | 0.. | 4 | 0 | 0 | — |
| —— Turkey, Fine | 9 | 10 | 0.. | 9 | 15 | 0 | — |
| —— Barbary | 5 | 5 | 0.. | 0 | 0 | 0 | — |
| —— Assafœtida | 10 | 0 | 0.. | 15 | 0 | 0 | — |
| —— Benjamin | 15 | 0 | 0.. | 60 | 0 | 0 | — |
| —— Gambogium | 24 | 10 | 0.. | 25 | 0 | 0 | — |
| —— Copal, scraped | 0 | 5 | 9.. | 0 | 6 | 0 | lb. |

| | £. | s. | d. | £. | s. | d. | per |
|---|---|---|---|---|---|---|---|
| Gum Galbanum | 24 | 0 | 0 to | 26 | 0 | 0 | C. |
| —— Guaiacum, from | 0 | 3 | 6.. | 0 | 5 | 0 | lb. |
| —— Mastic | 0 | 4 | 9.. | 0 | 5 | 0 | — |
| —— Myrrh | 16 | 16 | 0.. | 20 | 0 | 0 | C. |
| —— Olibanum | 8 | 0 | 0.. | 11 | 0 | 0 | — |
| —— Opponax | 80 | 0 | 0.. | 0 | 0 | 0 | — |
| —— Sandrac | 8 | 0 | 0.. | 8 | 5 | 0 | — |
| —— Senega | 6 | 15 | 0.. | 7 | 0 | 0 | — |
| —— Tragacanth | 23 | 10 | 0.. | 25 | 0 | 0 | — |
| Jalap | 0 | 5 | 0.. | 0 | 5 | 6 | lb. |
| Ipecacuanha | 0 | 12 | 0.. | 0 | 0 | 0 | — |
| Isinglass Book | 0 | 9 | 0.. | 0 | 0 | 0 | — |
| —— Leaf | 0 | 8 | 9.. | 0 | 0 | 0 | — |
| —— Long Staple | 0 | 9 | 6.. | 0 | 10 | 0 | — |
| —— Short Staple | 0 | 8 | 6.. | 0 | 0 | 0 | — |
| Manna Flakey | 0 | 5 | 2.. | 0 | 5 | 4 | — |
| —— Sicily in Sorts | 0 | 3 | 6.. | 0 | 0 | 0 | — |
| Musk China | 0 | 18 | 0.. | 1 | 0 | 0 | oz. |
| Nux Vomica | 2 | 0 | 0.. | 2 | 2 | 0 | C. |
| Oil of Vitriol | 0 | 0 | 3¾ | 0 | 0 | 4 | lb. |
| Opium, East-India | none | | .. | 0 | 0 | 0 | — |
| —— Turkey | 1 | 4 | 0.. | 1 | 4 | 6 | — |
| Pink Root | 2 | 1 | 0.. | 0 | 0 | 0 | — |
| Quicksilver | 0 | 4 | 6.. | 0 | 0 | 0 | — |
| Rhubarb, East-India | 0 | 6 | 6.. | 0 | 12 | 0 | — |
| —— Turkey | 0 | 18 | 0.. | 0 | 0 | 0 | — |
| Saffron, Spanish | 3 | 10 | 0.. | 3 | 14 | 0 | — |
| —— French | 2 | 4 | 0.. | 2 | 10 | 0 | — |
| Sago | 2 | 16 | 0.. | 4 | 0 | 0 | C. |
| Sal. Ammoniac | 10 | 10 | 0.. | 11 | 0 | 0 | — |
| Salop | 24 | 0 | 0.. | 25 | 0 | 0 | — |
| Sarsaparilla | 0 | 2 | 3.. | 0 | 4 | 6 | lb. |
| Sassafras | 1 | 10 | 0.. | 1 | 15 | 0 | T. |
| Scammony, Aleppo | 1 | 14 | 0.. | 1 | 16 | 0 | lb. |
| —— Smyrna | 0 | 18 | 0.. | 0 | 0 | 0 | — |
| Senna, Alexandria | 0 | 3 | 9.. | 0 | 0 | 0 | — |
| Seeds, Anni Alicant | 7 | 5 | 0.. | 8 | 0 | 0 | C. |
| —— Cummin | 4 | 10 | 0.. | 4 | 16 | 0 | — |
| —— Fenugreek | 1 | 15 | 0.. | 0 | 0 | 0 | — |
| Shellack | 5 | 0 | 0.. | 7 | 10 | 0 | — |
| Sticklack | 3 | 10 | 0.. | 7 | 0 | 0 | — |
| Snake Root | 0 | 3 | 9.. | 0 | 4 | 0 | lb. |
| Soap, Castile or Spanish | 9 | 0 | 0.. | 10 | 0 | 0 | C. |
| Spermaceti, refined | 0 | 2 | 2.. | 0 | 0 | 0 | lb. |
| Tamarinds, West-India | 5 | 5 | 0.. | 5 | 15 | 0 | C. |
| Tapioca, Lisbon | 0 | 0 | 9.. | 0 | 1 | 0 | lb. |
| Turmeric, Bengal | 4 | 10 | 0.. | 4 | 15 | 0 | C. |
| —— China | 6 | 10 | 0.. | 7 | 0 | 0 | — |
| —— West-India | 3 | 10 | 0.. | 4 | 4 | 0 | — |
| Verdigris, Wet | 0 | 3 | 6.. | 0 | 0 | 0 | lb. |
| —— Dry | 0 | 5 | 8.. | 0 | 5 | 10 | — |
| —— Crystallized | 0 | 8 | 9.. | 0 | 9 | 6 | — |
| Vitriol, Foreign white | 2 | 10 | 0.. | 0 | 0 | 0 | C. |

*Price of Vials per Gross.*—8 oz. 70s.—6 oz. 58s.—4 oz. 47s.—3 oz. 43s.—2 oz. 36s.—1 oz. 30s.—½ oz. 24s.

Lisbon Leeches, 30s. per hundred.—True French ditto, 55s.

Essential Salt of Lemons, 4s. 6d. per dozen.

METEOROLOGICAL

### METEOROLOGICAL REGISTER.

*From November the 25th, to December the 25th,* 1815.

**Kept by C. BLUNT, Philosophical Instrument Maker, No. 38, Tavistock-Street, Covent-Garden.**

| Moon. | Day. | Wind. | Barometrical Pressure. Max. | Min. | Mean. | Temperature. Max. | Min. | Mean. | |
|---|---|---|---|---|---|---|---|---|---|
| | 26 | N | 30·20 | 30·20 | 30·20 | 50 | 32 | 40·25 | Fair |
| | 27 | N | 30·19 | 30·01 | 30·127 | 48 | 32 | 39· | Fair |
| | 28 | N | 29·97 | 29·94 | 29·947 | 49 | 31 | 40· | Fair |
| | 29 | N | 30·07 | 30·02 | 30·022 | 50 | 33 | 39·5 | Rain |
| ● | 30 | NE | 29·84 | 29·72 | 29·797 | 51 | 35 | 40·75 | Fair |
| | 1 | NE | 29·95 | 29·79 | 29·835 | 49 | 33 | 39·5 | Fair |
| | 2 | NW | 30·11 | 30·07 | 30·092 | 51 | 34 | 39·5 | Fair |
| | 3 | W | 30· | 30· | 30· | 50 | 34 | 38·5 | Fair |
| | 4 | W | 29·79 | 29·73 | 29·755 | 49 | 35 | 38· | Fair |
| | 5 | SW | 29·91 | 29·41 | 29·665 | 50 | 35 | 38·75 | Fair |
| | 6 | SW | 29·42 | 29·38 | 29·405 | 50 | 34 | 39·75 | Rain |
| | 7 | W | 29·93 | 29·77 | 29·867 | 50 | 33 | 40· | Rain |
| ◐ | 8 | W | 29·97 | 29·96 | 29·965 | 48 | 31 | 37·75 | Rain |
| | 9 | W | 30·16 | 30·02 | 30·072 | 47 | 30 | 37·25 | Rain |
| | 10 | NW | 30· | 30· | 30· | 46 | 28 | 36·25 | Rain |
| | 11 | N | 30·31 | 30·30 | 30·02 | 47 | 26 | 35·5 | Fair |
| | 12 | N | 30·36 | 30·22 | 30·302 | 49 | 27 | 37· | Snow |
| | 13 | N | 30·20 | 30·13 | 30·157 | 51 | 25 | 35·25 | Snow |
| | 14 | N | 30·24 | 30·16 | 30·197 | 51 | 28 | 35·75 | Rain |
| | 15 | NW | 29·72 | 29·58 | 29·647 | 50 | 29 | 36·25 | Rain |
| ○ | 16 | W | 29·12 | 28·93 | 29·047 | 49 | 30 | 37· | Rain |
| | 17 | SW | 29·14 | 29·14 | 29·14 | 48 | 33 | 38·25 | Fair |
| | 18 | W | 29·42 | 29·35 | 29·385 | 49 | 28 | 37·25 | Fair |
| | 19 | NW | 29·56 | 29·50 | 29·527 | 48 | 30 | 36·25 | Snow |
| | 20 | WNW | 29·14 | 29·01 | 29·062 | 49 | 29 | 38·5 | Rain |
| | 21 | NW | 29·49 | 29·29 | 29·407 | 50 | 26 | 36· | Fair |
| | 22 | NW | 29·67 | 29·58 | 29·622 | 50 | 28 | 38· | Fair |
| ◑ | 23 | W | 29·69 | 29·49 | 29·60 | 52 | 28 | 36·5 | Fair |
| | 24 | W | 29·52 | 29·52 | 29·52 | 51 | 28 | 36·75 | Fair |
| | 25 | W | 29·52 | 29·52 | 29·52 | 48 | 26 | 35· | Fair |

RESULTS.

| | | | | | |
|---|---|---|---|---|---|
| Mean barometrical pressure of the month | | 29·848 | Mean temperature of the month | | 37·79625 deg. |
| Maximum 30·36, | wind at | N | Maximum 52, | wind at | W |
| Minimum 28·93 | ——— | W | Minimum 25, | ——— | N |

*Scale exhibiting the prevailing Winds during the Month.*

| N | NE | E | SE | S | SW | W | NW |
|---|---|---|---|---|---|---|---|
| 8 | 2 | 0 | 0 | 0 | 3 | 11 | 6 |

| | Mean barometrical pressure. | Mean temperature. |
|---|---|---|
| From the last quarter on the 23d Nov. to the new moon on the 30th. | 30·164 | 38·214 |
| ——— new moon on the 30th of Nov. to the first quarter on the 8th of Dec. | 29·802 | 29·347 |
| ——— first quarter on the 8th, to the full moon on the 16th | 30·045 | 36·375 |
| ——— full moon on the 16th, to the last quarter on the 23rd | 29·312 | 37·321 |

MONTHLY

### REPORT OF DISEASES.

The autumnal division of the year, to use the language of Sydenham, abounds quite as much with inflammatory complaints as the vernal; and this disposition to inflammation seems to increase from year to year. The more common complaints of this month are the exanthemata, particularly small-pox and scarlatina. Measles has not been so prevalent. Small-pox has been more destructive, compared with its prevalence, than at any period of the reporter's recollection, and more rife than for these last ten years. This cannot be the effect of inoculation, which no one, we presume, will have the hardiness to practise in the metropolis, since the decision at the Court of King's Bench. Again we must regret the inattention to vaccination.

All the fevers have been attended with strong local action, chiefly about the thorax, assuming the form of those violent pleurisies which Sydenham considered as confined to the spring. In one subject, not more than thirteen years old, but very well fed, it was found necessary, on the first day, to take eight ounces of blood, and exhibit strong cathartics. On the following, sixteen ounces were taken at two bleedings, yet, on the fourth day, carditis was evinced by every symptom of the pulse, the seat of the pain, the respiration, and the almost imperceptible pulsation of the heart. Sixteen ounces of blood were taken at a single bleeding, at the close of which operation the patient was slightly convulsed. From that time, however, the symptoms abated, and on the third day after it the patient became convalescent.

A child, three years old, in the family of a working artizan, not in an airy part of the town, was seized with croup, and effusion seemed to take place in the brain. He was leeched before the Reporter saw him; but the symptoms were so violent, that it was thought necessary immediately to take seven ounces of blood by cupping glasses, at the same time exhibiting calomel in very large and repeated doses. In the evening the breathing was so much relieved as to give every hope of recovery, but the faculties remained dull for four days more, during which the calomel was continued in smaller doses. At the end of that time he recovered his senses, and his speech imperfectly. He is now convalescent.

The practice of bandaging in rheumatism gains ground. We hope it will not, like other useful remedies, be so hacknied as to be brought into disgrace for not proving universally successful.

Catarrhs are somewhat abating in frequency and severity; but the winter asthmas are beginning to interrupt the conviviality of the season.

